# Nipple-areolar complex (NAC) or skin flap ischemia necrosis post nipple-sparing mastectomy (NSM)—analysis of clinicopathologic factors and breast magnetic resonance imaging (MRI) features

**DOI:** 10.1186/s12957-023-02898-x

**Published:** 2023-01-25

**Authors:** Hung-Wen Lai, Yi-Yuan Lee, Shou-Tung Chen, Chiung-Ying Liao, Tsung-Lin Tsai, Dar-Ren Chen, Yuan-Chieh Lai, Wen-Pin Kao, Wen-Pei Wu

**Affiliations:** 1 Endoscopic & Oncoplastic Breast Surgery Center, Changhua Christian Hospital, Changhua, Taiwan; 2 Division of General Surgery, Department of Surgery, Changhua Christian Hospital, Changhua, Taiwan; 3 Comprehensive Breast Cancer Center, Changhua Christian Hospital, Changhua, Taiwan; 4 Minimal Invasive Surgery Research Center, Changhua Christian Hospital, Changhua, Taiwan; 5 Kaohsiung Medical University, Kaohsiung, Taiwan; 6 Division of Breast Surgery, Yuanlin Christian Hospital, Yuanlin, Taiwan; 7 School of Medicine, Chung Shan Medical University, Taichung, Taiwan; 8 School of Medicine, National Yang Ming Chiao Tung University, Taipei, Taiwan; 9 Department of Public Health, China Medical University, Taichung, Taiwan; 10 Department of Radiology, Changhua Christian Hospital, Changhua, Taiwan; 11 Department of Biomedical Imaging and Radiological Sciences, National Yang Ming Chiao Tung University, Taipei, Taiwan; 12 Division of Plastic Surgery, Department of Surgery, Changhua Christian Hospital, Changhua, 500 Taiwan

**Keywords:** Nipple-sparing mastectomy (NSM), Nipple-areola complex (NAC), MRI, Ischemia necrosis, Robotic-assisted nipple-sparing mastectomy (R-NSM), Endoscopic-assisted nipple-sparing mastectomy (E-NSM), Skin flap

## Abstract

**Background:**

The purpose of this study is to identify clinicopathologic factors and/or preoperative MRI vascular patterns in the prediction of ischemia necrosis of the nipple-areola complex (NAC) or skin flap post nipple-sparing mastectomy (NSM).

**Methods:**

We performed a retrospective analysis of 441 NSM procedures from January 2011 to September 2021 from the breast cancer database at our institution. The ischemia necrosis of NAC or skin flap was evaluated in correlation with clinicopathologic factors and types of skin incision. Patients who received NSM with preoperative MRI evaluation were further evaluated for the relationship between vascular pattern and the impact on ischemia necrosis of NAC or skin flap.

**Results:**

A total of 441 cases with NSM were enrolled in the current study, and the mean age of the cases was 49.1 ± 9.8 years old. A total of 41 (9.3%) NSM procedures were found to have NAC ischemia/necrosis. Risk factors were evaluated of which old age, large mastectomy specimen weight (> 450 g), and peri-areola incision were identified as predictors of NAC necrosis. Two-hundred seventy NSM procedures also received preoperative MRI, and the blood supply pattern was 18% single-vessel type and 82% double-vessel pattern. There were no correlations between MRI blood supply patterns or types of skin flap incisions with ischemia necrosis of NAC. There were also no correlations between blood loss and the pattern or size of the blood vessel.

**Conclusion:**

Factors such as the type of skin incision, age, and size of mastectomy weight played an important role in determining ischemia necrosis of NAC; however, MRI vascular (single or dual vessel supply) pattern was not a significant predictive factor.

**Supplementary Information:**

The online version contains supplementary material available at 10.1186/s12957-023-02898-x.

## Synopsis

Nipple-areola complex (NAC) or skin flap ischemia/necrosis is one of the major complications of nipple-sparing mastectomy (NSM). This study aims to identify clinicopathologic risk factors or MRI features predictive of ischemia/necrosis of the NAC or skin flap. Old age, large mastectomy specimen weight (> 450 g), and peri-areola incision were identified as known risk factors for NAC ischemia/necrosis. There were however no correlations between MRI blood supply pattern, skin incision placement, blood loss, or size of blood vessel with ischemia necrosis of NAC.

## Introduction

Nipple-sparing mastectomy (NSM) has gradually become one of the standard surgical treatment options for breast cancer patients indicated for mastectomy without apparent nipple-areolar complex (NAC) involvement due to its better cosmetic results and acceptable oncologic outcome [1–6]. Studies have shown that preserving NAC did not significantly increase the risk of local recurrence compared to skin-sparing mastectomy, and oncologic safety is comparable to traditional mastectomy [7–10].

To reduce locoregional recurrence, NSM techniques involve the removal of glandular and ductal tissue from beneath the NAC, which may affect NAC vascularity and result in NAC ischemia or necrosis. NAC or skin flap ischemia/necrosis is one of the important complications of NSM [11–14] with the incidence rate varying from 12.2 to 64.1% as it may result in suboptimal esthetic results and increased patients’ anxiety [15]. According to a recent meta-analysis, partial or complete nipple necrosis occurred in around 15% of patients who received NSM, which accounts for the majority of overall complications [16]. Risk factors of ischemia/necrosis of the NAC were identified from some studies [17–21], which included body mass index (BMI) greater than 30 kg/m^2^, diabetes, heavy smoking, breast sagging, use of steroids, personal medical history, massive removal of breast tissue, and type of incisions. With the improved knowledge of ischemia necrosis of NAC and refinement of surgical technique of NSM, NAC ischemia/necrosis events had decreased, but it still remained an important complication to avoid at all costs, if possible.

Rusby et al. [22] suggested that the type of incision will affect the risk of NAC necrosis, and incisions around the areola had a higher risk than incisions placed further away from the areolar. Bahl et al. [23] proposed that the pattern of blood supply as shown on preoperative breast magnetic resonance imaging (MRI) could be predictive of the risk of postoperative ischemia necrosis of NAC. A double blood vessel supply has been shown to reduce the risk of ischemic necrosis of NAC. In contrast to types of skin incision, which had been confirmed from previous evidence [11, 24–27], studies looking at preoperative MRI blood vessel pattern as a predictive factor of NAC ischemia necrosis [23] were lacking.

The aim of the current study is to identify and validate the potential risk factors of NAC and/or skin flap ischemia necrosis, such as wound incision type, BMI, and breast size. The findings of preoperative breast MRI vascular pattern (single versus double vessel supply) would also be analyzed to assess for any correlations with NAC ischemia necrosis.

## Materials and methods

### Patient selection and data collection

To evaluate and validate the potential risk factors associated with NAC ischemia necrosis, patients who underwent NSM from January 2011 to September 2021 were identified from a prospectively maintained breast cancer database at Changhua Christian Hospital (CCH), a tertiary medical center in Central Taiwan. Those patients whose clinicopathologic factors, information regarding the types of skin incision, postoperative NAC, or skin flap ischemia necrosis status could not be clearly identified were excluded from this study.

The collected data was obtained from the medical records of these patients, including age, BMI, pathologic reports, complications, ischemia necrosis of the NAC or skin flap, the location of the surgical incision, and status of follow-up. Patients with preoperative breast MRI were further analyzed in terms of the pattern of blood supply (single vessel or double vessel) of the breast and the diameter of the vessel. The pattern of blood supply and diameter of the blood vessel was correlated with ischemia necrosis of NAC or skin flap condition to determine if these factors were predictive of NAC or skin flap necrosis. Combined blood supply pattern (single versus double vessel) in different types of skin incisions was also assessed to ascertain its impact on ischemia necrosis of NAC. A literature review of reported studies [12, 19, 20, 23, 25–35] regarding risk factors and incidence of NAC ischemia necrosis was also performed in this study.

**Fig. 1:**
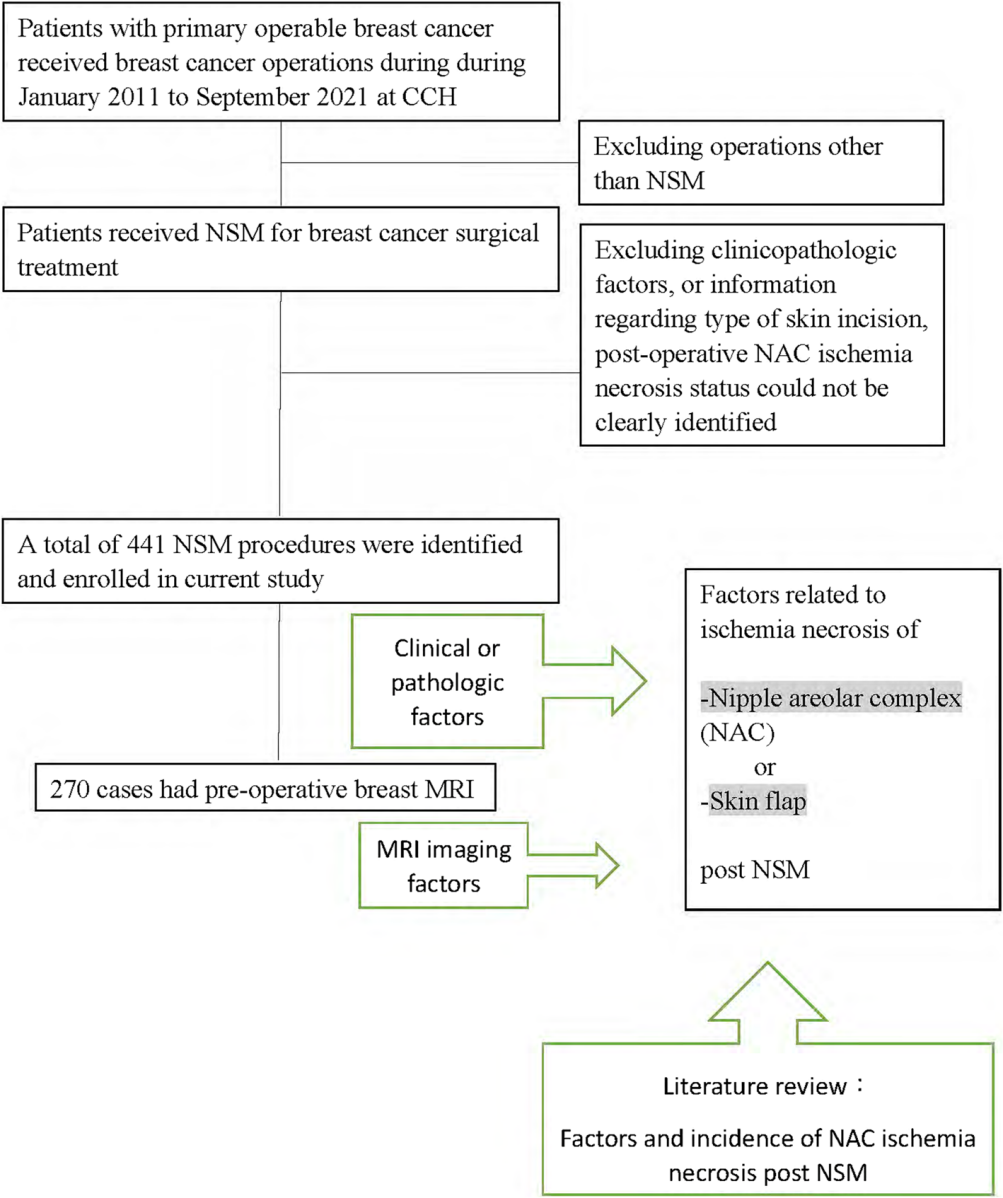
Flow chart of study design

According to the inclusion and exclusion criteria, a total of 441 NSM procedures were identified and enrolled in the current study, and among them, 270 cases had preoperative breast MRI. The study was approved by the Institutional Review Board of the CCH (CCH IRB no. 141224 & 201242), and all patients consented to the study. The study design and patients’ flow chart were shown in Fig. 1.

### Grading of nipple-areolar complex (NAC) ischemia and necrosis

**Fig. 2:**
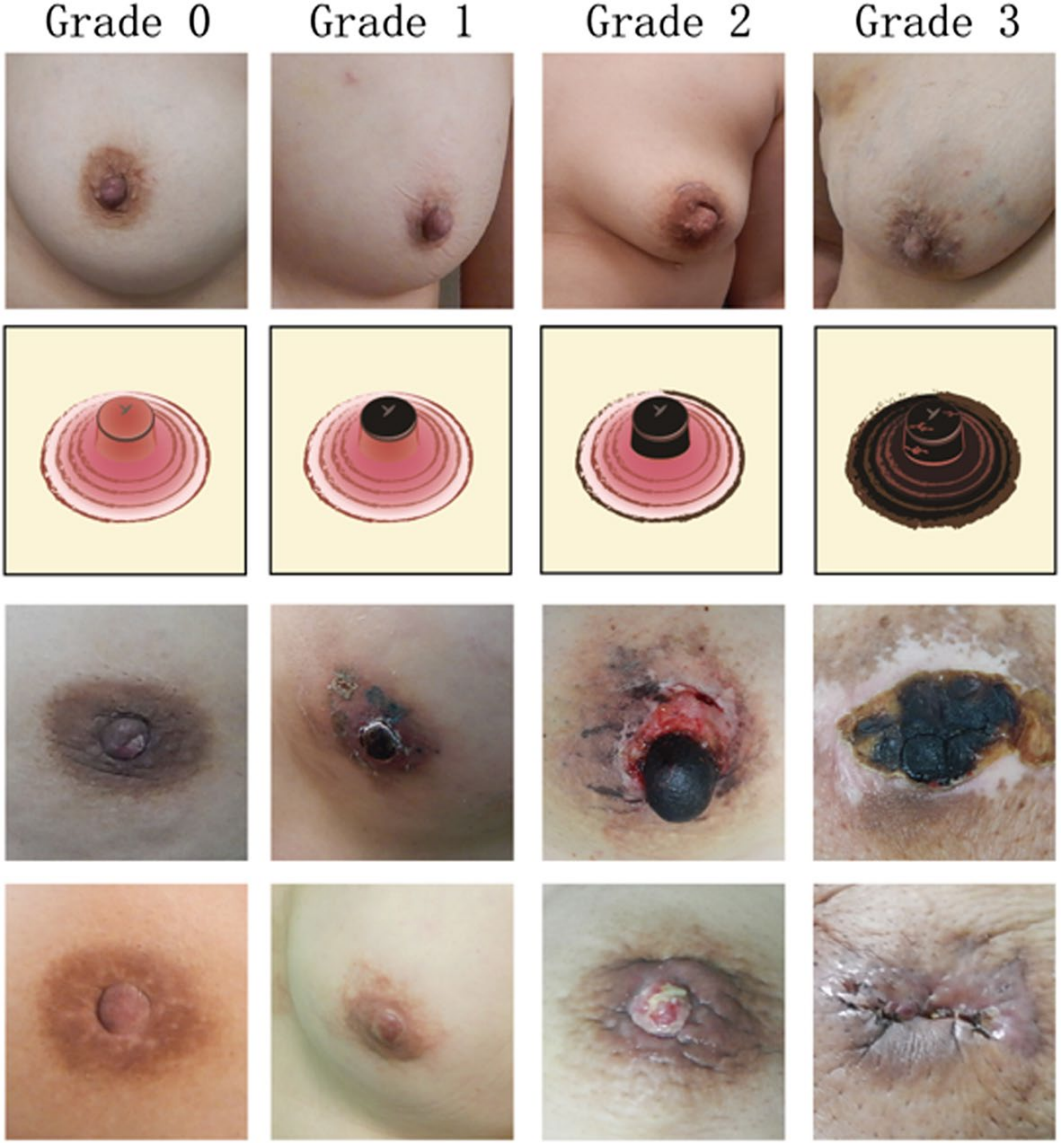
Figure illustrations of nipple-areolar complex (NAC) necrosis grading. **a** Grade 0 NAC ischemia necrosis: the nipple is normal, and there is no necrosis of NAC at all. No ischemia necrosis change of NAC post NSM. **b** Grade 1 NAC ischemia necrosis: NAC underwent transient ischemia injury with slightly gangrene change, which was reversible after conservative treatment. There would be mild (< 25%) to no volume loss of the nipple after recovery. **c** Grade 2 NAC ischemia necrosis: NAC underwent moderate ischemia necrosis, which was irreversible, and eventually leading to loss of around 50% (25–75%) of the original volume. **d** Grade 3 NAC ischemia necrosis: the most severe form of ischemia necrosis of NAC post NSM. The nipple and/or areolar complex suffered from nearly > 75% to complete (100%) loss of nipple volume and eventually leading to surgical excision of nipple or NAC, which left no apparent nipple or NAC tissue

In this study, nipple ischemia necrosis was divided into 4 grades, designated as grades 0, 1, 2, and 3 (Fig. 2). In grade 0 NAC ischemia/necrosis, the nipple is normal; there is no necrosis of NAC at all. Grade 1 NAC ischemia referred to transient ischemia injury with slightly ischemic change, which was reversible after conservative treatment. There would be minimal (< 25%) to no nipple volume loss after recovery. Grade 2 NAC ischemia necrosis referred to irreversible moderate ischemia necrosis, which eventually leads to a loss of around 50% (25–75%) of the original volume. Grade 3 NAC ischemia necrosis was the most severe form of necrosis injury. The NAC suffered from near (> 75%) to complete (100%) loss of volume and eventually leading to surgical excision or total loss of the NAC tissue.

To evaluate the risk factors associated with NAC ischemia necrosis, those patients who suffered from grade 2 or grade 3 NAC ischemia necrosis (Fig 2) were recorded as events of NAC necrosis in the current study.

### Type of skin flap incisions

NSMs were performed via various skin incisions. Skin incisions were divided into the following categories for analytic purposes: upper outer incision (radial incision), the peri-areolar-related incision (with or without axillary incision), single axillary incision, and infra-mammary + axillary incisions (Supplementary File 1). In our study, the NSM procedures with single axillary incision were performed with endoscopic-assisted or robotic-assisted NSMs [36].

### Body mass index (BMI) category

BMI was calculated as weight in kilograms divided by height in meters squared (*BMI* = kg/m^2^). Using the Taiwanese definition, BMI was categorized into four groups: underweight (*BMI* < 18.5), normal (BMI of 18.5 to 24), overweight (BMI of 24.1 to 26.9), and obese (*BMI* ≥ 27). Patients were further categorized into 2 groups, which included non-obese (*BMI* < 27) and obese (*BMI* ≥ 27), for analytic purposes in the current study.

### Magnetic resonance imaging (MRI) and protocol

MR imaging was performed with a Siemens MAGNETOM Verio 3.0 Tesla MRI machine. All patients were imaged in the prone position with both breasts placed into a dedicated 16-channel breast coil. MR imaging protocols included the following: bilateral axial turbo-spin-echo fat-suppressed T2-weighted imaging (TR/TE 4630/70 ms; field of view 320 mm; slice thickness 3 mm; number of excitations 1), axial turbo-spin-echo T1-weighted imaging (TR/TE 736/9.1 ms; field of view 320 mm; slice thickness 3 mm; number of excitations 1), and diffusion-weighted imaging (TR/TE 5800/82 ms; field of view 360 mm; slice thickness 3 mm, with *b*-values of 0, 400, and 800 s/mm^2^). Dynamic contrast-enhanced MR images (DCE-MRI) were obtained with a three-dimensional fat-suppressed volumetric interpolated breath-hold examination (VIBE) sequence with parallel acquisition once before and five times after a bolus injection of gadobenate dimeglumine (0.1 mmol/kg). Both breasts were examined in the transverse plane at 60 s intervals in each phase of the dynamic studies. The dynamic MRI parameters were as follows: TR/TE 4.36/1.58 ms, field of view 320 mm, and slice thickness 1 mm. The whole breast MRI readings were carried out by two experienced, board-certified breast radiologists (WPW and CYL).

Breast MRI had been part of preoperative evaluation for breast cancer patients diagnosed and treated at CCH. Patients who underwent preoperative breast MRI were retrospectively evaluated with regard to blood flow pattern. To understand the distribution and blood supply of blood vessels around the areola, MRI is used, and the blood supply pattern is divided into the dual blood supply and single blood supply. The diameter of the blood vessel was also divided into less than 1 mm, equal to 1 mm, and greater than 1 mm. The interpretation of blood vessel type and pattern was done by an experienced breast imaging radiologist (WPW) with more than 10 year’s experience in breast MRI imaging.

### Statistical analysis

Data are expressed as mean ± standard deviation and compared using the parametric two-sample *t*-test. Baseline data were analyzed using chi-square tests (categorical data) or Student’s *t*-test (continuous data). Associations between nipple ischemia necrosis and risk factors of patients were analyzed using the chi-square test. Multivariate logistics was performed to assess the association between NAC and/or skin flap ischemia necrosis and risk factors. Results were considered statistically significant if the two-tailed *p*-value was < 0.05 for all tests. Statistical analyses were performed using SAS 9.4 version (SAS Inc., Cary, NC, USA) by a statistician (YYL).

## Results

According to the inclusion and exclusion criteria, a total of 441 NSM procedures were enrolled in the current study. The mean age was 49.1 ± 9.8 years old, and breast reconstructions were performed in 83.7% (369/441). Among the 369 NSM cases with breast reconstructions, 321 received gel implants, 9 tissue expanders, 33 TRAM flaps, 4 LD flaps, and 2 LD flaps + implants. Types of skin incisions (Supplementary File 1) were 83 (18.9%) upper outer oblique (radial) incisions, 107 (24.3%) peri-areolar-related incisions, 243 (55.2%) single axillary incisions, and 7 (1.6%) infra-mammary + axillary incisions. Among these 441 NSM procedures, NAC ischemia necrosis grading was 84.1% (371/441) grade 0, 6.6% (29/441) grade 1, 8.4% (37/441) grade 2, and 0.9% (4/441) grade 3 (Table 1, Fig. 2). According to NAC ischemia necrosis criteria used in the current study, about 9.3% (41/441) of NSM procedures were graded as having NAC necrosis events. The demographic and clinical characteristics of the patients were summarized in Table 1.

**Table 1:** Clinicopathologic manifestations of 441 nipple-sparing mastectomy procedures enrolled in current study

*n* = 441	Mean (sd), *n* (%)
**Age, y**	49.1 ± 9.8
**BMI**	
< 27	384 (87.1)
≥ 27	57 (12.9)
**Location**	
Left	219 (49.7)
Right	222 (50.3)
**Surgery type**	
Convention	106 (24.0)
Endoscopy	242 (54.9)
Robot	93 (21.1)
**Reconstruction**	
Yes	369 (83.7)
No	72 (16.3)
**Reconstruction method (*n*** **= 369)**	
Tissue expander	9 (2.4)
Gel implant	321 (87.0)
TRAM flap	33 (8.9)
LD flap + gel implant	2 (0.5)
LD flap	4 (1.1)
**Type of skin incisions** (N/A = 1)	
Upper outer oblique incisions	83 (18.9)
Peri-areolar incisions	107 (24.3)
Single axillary incisions	243 (55.2)
Infra-mammary + axillary incisions	7 (1.6)
**Pathology tumor size, cm**	2.6 ± 2.3
**Grade** (N/A = 102)	
I	65 (19.2)
II	204 (60.2)
III	70 (20.6)
**Clinical stage** (N/A = 132)	
0	76 (24.6)
I	59 (19.1)
II	161 (52.1)
III	13 (4.2)
**Subtype** (N/A = 113)	
Luminal A	152 (46.3)
Luminal B1	81 (24.7)
Luminal B2	40 (12.2)
HER-2 (+)	26 (7.9)
TNBC	29 (8.8)
**Lymph node stage** (N/A = 61)	
N0	271 (71.3)
N1	84 (22.1)
N2	22 (5.8)
N3	3 (0.8)
**Surgical ALN staging method** (N/*A* = 129)	
ALND	24 (6.4)
SLNB	281 (74.7)
SLNB+ALND	71 (18.9)
**Lymph node metastasis** (N/A = 61)	
Yes	109 (28.8)
No	270 (71.2)
**Stage** (N/A = 80)	
0	82 (22.7)
I	95 (26.3)
II	151 (41.8)
III	33 (9.1)
**ER** (N/A = 86)	
Positive	289 (81.4)
Negative	66 (18.6)
**PR** (N/A = 89)	
Positive	246 (69.9)
Negative	106 (30.1)
**HER-2** (N/A = 134)	
Positive	83 (27.0)
Negative	224 (73.0)
**Ki-67 (%)** (N/A = 181)	
≦ 14	103 (39.6)
> 14	157 (60.4)
**Pathology** (N/A = 11)	
DCIS	85 (19.8)
IDC	239 (55.6)
ILC	14 (3.3)
LCIS	4 (0.9)
Other	88 (20.5)
**NAC ischemia necrosis**	
0	371 (84.1)
1	29 (6.6)
2	37 (8.4)
3	4 (0.9)
**Skin flap necrosis**	
0	417 (94.6)
1	22 (5.0)
2	2 (0.5)

*BMI* Body mass index, *TRAM flap* Transverse rectus abdominal myocutaneous flap, *LD flap* Latissimus dorsi flap, *ALN* Axillary lymph node, *ALND* Axillary lymph node dissection, *SLNB* Sentinel lymph node biopsy, *NAC* Nipple areolar complex

Risk factors associated with NAC necrosis were evaluated, and the mean age of patients with NAC necrosis was 53 ± 10.7 years old compared with patients without NAC necrosis (48.7 ± 9.6), which tend to be older (*p* < 0.01). Patients with specimen weights of more than 450 g had a higher incidence of NAC ischemia necrosis (16.9%, 14/83) than those (4.3%, 3/70) with less than 180 grams (*p* = 0.03, Table 2). In terms of skin incision analysis, patients with peri-areola incisions had a higher incidence of nipple necrosis (19.6%, 21/107) compared to patients with single axillary incision (6.2%, 15/243) or upper outer oblique (radial) incision (6%, 5/83, *p*-value < 0.01). Similar findings were also observed in NAC or skin necrosis, which showed that older age, larger specimen weight, and wound incision type were significant risk factors. Single axillary incision (7.4%) was associated with lower NAC or skin flap necrosis than upper oblique (radial) incision (14.4%) or per-areolar incision (22.4%, *p* < 0.01). Factors related to ischemia necrosis of NAC and/or skin flap were summarized in Table 2.

**Table 2:** Factors associated with nipple-areolar complex (NAC) and skin necrosis

Characteristics		Nipple-areola complex Ischemia necrosis			Nipple-areola complex Ischemia necrosis or skin flap necrosis		
	Total (***n*** = 441)	Yes (***n*** = 41)	No (***n*** =4 00)	***p***-value	Yes (***n*** = 54)	No (***n*** = 387)	***p***-value
**Age**	49.1 ± 9.8	53 ± 10.7	48.7 ± 9.6	< 0.01	51.2 ± 10.7	48.8 ± 9.6	0.09
**BMI**	23 ± 3.5	23.9 ± 4.3	23.0 ± 3.4	0.16	23.9 ± 4.1	22.9 ± 3.4	0.09
**BMI**				0.19			0.08
BMI < 27	384 (87.1)	33 (80.5)	351 (87.8)		43 (11.2)	341 (88.8)	
BMI ≥ 27	57 (12.9)	8 (19.5)	49 (12.3)		11 (19.3)	46 (80.7)	
**Reconstruction**				0.10			0.02
Yes	369 (83.7)	38 (92.7)	331 (82.8)		51 (94.4)	318 (82.2)	
No	72 (16.3)	3 (7.3)	69 (17.3)		3 (5.6)	69 (17.8)	
**Reconstruction type**				0.62			0.57
Tissue expander	9 (2.4)	0 (0.0)	9 (2.7)		0(0.0)	9 (2.83)	
Gel implant	321 (87.0)	36 (94.7)	285 (86.1)		45 (88.24)	276 (86.79)	
TRAM Flap	33 (8.9)	2 (5.3)	31 (9.4)		6 (11.76)	27 (8.49)	
LD flap + gel implant	2 (0.5)	(0.0)	2 (0.6)		0 (0.0)	2 (0.63)	
LD flap	4 (1.1)	(0.0)	4 (1.2)		0 (0.0)	4 (1.26)	
**Specimen**	337.6 ± 207.3	427.2 ± 235.5	328.2 ± 202.2	< 0.01	432.8 ± 224.1	324.0 ± 201.5	< 0.01
**Specimen**				0.03			< 0.01
< 180	70 (16.2)	3 (7.3)	67 (17.1)		3 (5.6)	67 (17.7)	
180–320	174 (40.3)	12 (29.3)	162 (41.4)		16 (29.6)	158 (41.8)	
320–450	105 (24.3)	12 (29.3)	93 (23.8)		15 (27.8)	90 (23.8)	
> 450	83 (19.2)	14 (34.1)	69 (17.6)		20 (37.0)	63 (16.7)	
**Tumor-nipple distance**	3.2 ± 1.8	3.4 ± 1.6	3.1 ± 1.8	0.41	3.7 ± 1.7	3.1 ± 1.8	0.04
**Neoadjuvant CT**				0.04			0.13
Yes	46 (15.2)	1 (3.0)	45 (16.7)		3 (7.3)	43 (16.5)	
No	256 (84.8)	32 (97.0)	224 (83.3)		38 (92.7)	218 (83.5)	
**ER**				0.62			0.78
Positive	289 (81.4)	29 (78.4)	260 (81.8)		40 (80.0)	209 (69.2)	
Negative	66 (18.6)	8 (21.6)	58 (18.2)		10 (20.0)	93 (30.8)	
**PR**				0.96			0.49
Positive	246 (69.9)	26 (70.3)	220 (69.8)		37 (74.0)	219 (85.0)	
Negative	106 (30.1)	11 (29.7)	95 (30.2)		13 (26.0)	93 (87.7)	
**HER-2**				0.94			0.84
Positive	83 (27)	9 (26.5)	74 (27.1)		13 (28.3)	70 (26.8)	
Negative	224 (73)	25 (73.5)	199 (72.9)		33 (71.7)	191 (73.2)	
**Stage**				0.58			0.28
Stage 0	82 (22.7)	8 (20.5)	74 (23)		9 (17.3)	73 (23.6)	
Stage 1	95 (26.3)	9 (23.1)	86 (26.7)		12 (23.1)	83 (26.9)	
Stage 2	151 (41.8)	20 (51.3)	131 (40.7)		28 (18.5)	123 (39.8)	
Stage 3	33 (9.1)	2 (5.1)	31 (9.6)		28 (53.8)	30 (9.7)	
**Skin incision**				< 0.01			< 0.01
Upper outer oblique (radial)	83 (18.9)	5 (12.2)	78 (19.5)		12 (22.2)	71 (18.4)	
Peri-areolar	107 (24.3)	21 (51.2)	86 (21.6)		24 (44.4)	83 (21.5)	
Single axillary	243 (55.2)	15 (36.6)	228 (57.1)		18 (33.3)	225 (58.3)	
Infra-mammary + axillary	7 (1.6)	0 (0.0)	7 (1.8)		0 (0.0)	7 (1.8)	

The related risk factors associated with ischemia necrosis of NAC and/or skin flap were further analyzed with univariate and multivariate logistic regression (Table 3). In multivariate logistic regression analysis, compared to the upper outer (radial) incision, the peri-areolar-related incision was significantly associated with higher NAC ischemia necrosis (odd ratio = 5.33, *p* < 0.01). Increasing age was associated with a higher risk of NAC ischemia necrosis (odds ratio = 1.04, *p* = 0.02). Compared with small breast (mastectomy specimen weight < 180 g), larger breast (mastectomy specimen weight > 450 g) was significantly associated with a higher risk of NAC ischemia necrosis (odds ratio = 4.6, *p* = 0.03) or NAC-or-skin flap necrosis (odds ratio = 6.99, *p* < 0.01, Table 3).

**Table 3:** Association between nipple-areola complex ischemia necrosis and risk factors by the logistic regression (*n* = 441)

**Univariate logistic regression**						
	**Nipple-areola complex ischemia necrosis**			**Nipple-areola complex ischemia necrosis or skin flap necrosis**		
	Odds ratio	95% *CI*	*p*-value	Odds ratio	95% *CI*	*p*-value
**Skin incision**						
Upper outer oblique	1	-	-	1	-	-
Peri-areolar	3.81	1.42–10.98	< 0.01	1.71	0.80–3.67	0.17
Single axillary	1.03	0.36–2.92	0.96	0.47	0.22–1.03	0.06
Infra-mammary + axillary	-	-	0.99	-	-	0.99
**Age**	1.04	1.01–1.07	0.01	1.02	1.00–1.05	0.10
**Specimen**						
< 180	1	-	-	1	-	-
180–320	1.63	0.44–5.95	0.46	2.22	0.63–7.89	0.22
320–450	2.89	0.78–10.68	0.11	3.74	1.04–13.44	0.04
> 450	4.46	1.23–16.24	0.02	6.99	1.98–24.70	< 0.01
**Multivariate logistic regression**						
	Odds ratio	95% *CI*	*p*-value	Odds ratio	95% *CI*	*p*-value
**Skin incision**						
Upper outer oblique	1	-	-	1	-	-
Peri-areolar	5.33	1.81–15.67	< 0.01	2.30	1.02–5.17	0.04
Single axillary	1.22	0.41–3.60	0.72	0.56	0.25–1.26	0.16
Infra-mammary + axillary	-	-	0.99			
**Age**	1.04	1.01–1.08	0.02	1.02	0.99–1.05	0.21
**Specimen**						
< 180	1	-	-	1	-	-
180–320	1.36	0.36–5.22	0.65	2.15	0.59–7.86	0.25
320–450	2.28	0.58–8.89	0.24	3.45	0.92–12.910	0.07
> 450	4.60	1.20–17.71	0.03	6.99	1.91–25.64	< 0.01

**Fig. 3:**
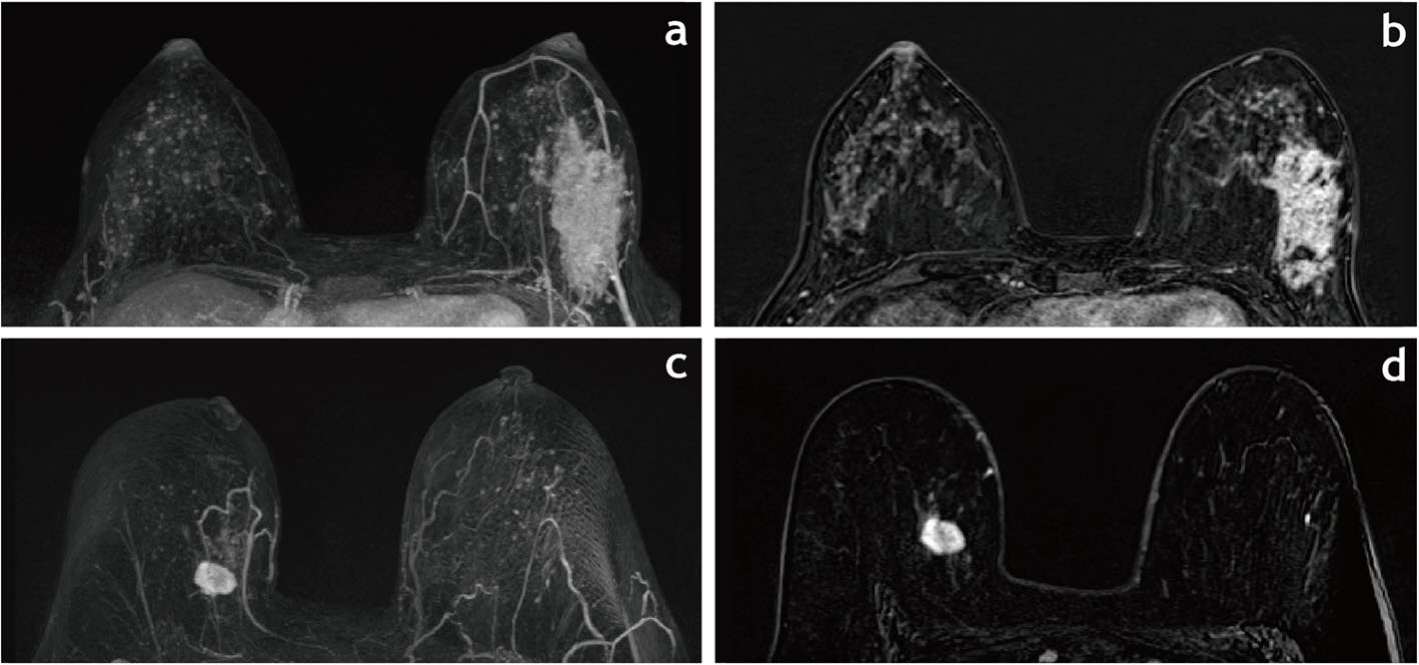
Vascular patterns on breast MRI with dual blood supply (**a**–**b**) and single blood supply (**c**–**d**). **a** and **b** A 36-year-old female with invasive ductal carcinoma and extensive ductal carcinoma in situ in the left breast underwent nipple-sparing mastectomy (NSM). Axial maximum intensity projection image (**a**) demonstrates dual blood supply with codominance of the medial and lateral vessels. **b** Early-subtracted dynamic contrast-enhanced T1-weighted sequence shows regional distribution, non-mass enhancement of the left breast, with heterogenous contrast enhancement and irregular shape. **c** and **d** A 61-year-old female with invasive carcinoma in the right breast underwent NSM. Axial maximum intensity projection image (**c**) demonstrates single blood supply. **d** Early-subtracted dynamic contrast-enhanced T1-weighted sequence shows a round mass in the lower-outer quadrant of the right breast. Measurement of the lesion: 22 mm. Histological examination of the lesion confirmed invasive ductal carcinoma (diameter 23 mm)

Of the 441 NSM patients, 270 had received preoperative MRI evaluation. Among these patients, the blood supply pattern was 18% (47/261) with single vessel blood supply and 82% (214/261) with double blood vessel supply (Fig 3). The blood vessel diameter was > 1 mm in 61.7% (161/261), 1 mm in 17.2% (45/261), and < 1 mm in 21.1% (55/261) of patients with preoperative MRI. In these 270 NSM procedures, NAC ischemia necrosis was found in 9.6% (26/270) of NSM procedures.

In patients who received preoperative breast MRI evaluation, the incidence of NAC necrosis in single blood vessel pattern was 4.3% (2/47) and 10.8% (23/214) in double blood vessel cases (*p*-value = 0.17, Table 4). The incidence of NAC necrosis was 17.8% (8/45) in vessel diameter of 1 mm, 9.9% (16/161) in > 1 mm diameter, and 1.8% (1/55) in vessel < 1 mm (*p* = 0.03). In these 270 cohorts of patients, skin incision type remained an important factor related to NAC necrosis, with 4.6% (3/66) in the upper outer quadrant (radial) incision group, 23% (17/74) in the peri-areolar related incision, and 4.8% (6/126) in single axillary incision (*p* < 0.01).

**Table 4:** Nipple-areola complex ischemia necrosis and/or skin flap necrosis in patients with preoperative breast MRI

**Characteristics**	**Total (*n*** **= 270)**	**Nipple-areola complex ischemia necrosis**			**Nipple-areola complex ischemia necrosis or skin flap necrosis**		
		**Yes (*n*** **= 26)**	**No (*n*** **= 244)**	***p*-value**	**Yes (*n*** **= 35)**	**No (*n*** **= 235)**	***p*-value**
**Skin incision**				< 0.01			< 0.01
Upper outer oblique (radial)	66 (24.4)	3 (11.5)	63 (25.8)		6 (9.1)	60 (90.9)	
Peri-areolar	74 (27.4)	17 (65.4)	57 (23.4)		20 (27.0)	54 (73.0)	
Single axillary	126 (46.7)	6 (23.1)	120 (49.2)		9 (7.1)	117 (92.9)	
Infra-mammary + axillary	4 (1.5)	0 (0.0)	4 (1.6)		0 (0.0)	4 (100.0)	
**Blood supply** (*NA* = 9)				0.17			0.35
Single	47 (18.0)	2 (8.0)	45 (19.1)		4 (12.1)	43 (18.9)	
Dual	214 (82.0)	23 (92.0)	191 (80.9)		29 (87.9)	185 (81.1)	
**Blood supply** (upper outer oblique)				0.42			0.58
Single	11 (16.9)	0 (0.0)	11 (17.7)		0 (0.0)	11 (18.6)	
Dual	54 (83.1)	3 (100.0)	51(82.3)		6 (100.0)	48 (81.4)	
**Blood supply** (peri-areolar)				0.13			0.15
Single	21 (28.8)	2 (9.5)	19 (90.5)		3 (15.8)	18 (33.3)	
Dual	52 (71.2)	14 (26.9)	51 (82.3)		16 (84.2)	36 (66.7)	
**Blood supply** (single axillary)				1			1
Single	15 (12.6)	0 (0.0)	15 (13.3)		1 (12.5)	14 (12.6)	
Dual	104 (87.4)	6 (100.0)	98 (86.7)		7 (87.5)	97 (87.4)	
**Blood supply** (infra-mammary + axillary)				-			-
Single	0 (0.0)	0	0 (0.0)		0	0 (0.0)	
Dual	4 (100.0%)	0	4 (100.0)		0	4 (100.0)	
**Blood diameter** (*NA* = 9)				0.03			0.15
< 1 mm	55 (21.1)	1 (4.0)	54 (22.9)		3 (9.1)	52 (22.8)	
= 1 mm	45 (17.2)	8 (32.0)	37 (15.7)		8 (24.2)	37 (16.2)	
> 1 mm	161 (61.7)	16 (64.0)	145 (61.4)		22 (66.7)	139 (61.0)	
Analyze the correlation between MRI vascular pattern and nipple-areola necrosis							
	**Nipple-areola complex ischemia necrosis**						***p*-value**
	**Total (*n*** **= 261)**	**0 (*n*** **= 218)**	**1 (*n*** **= 18)**	**2 (*n*** **= 22)**	**3 (*n*** **= 3)**		
**Blood supply**							0.19
Single	47 (18.0)	39 (17.9)	6 (33.3)	2 (9.1)	0 (0.0)		
Dual	214 (82.0)	179 (82.1)	12 (66.7)	20 (90.9)	3 (100.0)		
**Blood diameter**							0.25
< 1	55 (21.1)	51 (23.4)	3 (16.7)	1 (4.5)	0 (0.0)		
= 1	45 (17.2)	34 (15.6)	3 (16.7)	7 (31.8)	1 (33.3)		
> 1	161 (61.7)	133 (61.0)	12 (66.7)	14 (63.6)	2 (66.7)		
Total 261 patients, excluding NAC ischemia grade 0 = 218							
Excluding 2 cases whose detailed information could not be traced							
Analyze the correlation between MRI vascular patterns and blood loss							
	**Blood supply**						***p*-value**
	**Total (*n*** **= 249)**	**Single (*n*** **= 44)**		**Dual (*n*** **= 205)**			0.49
**Blood loss total**	94.3 ± 80.1	101.9 ± 77.7		92.7 ± 80.7			
	**Blood diameter**						
	**Total (*n*** **= 249)**	**< 1 (*n*** **= 52)**	**= 1 (*n*** **= 41)**		**> 1 (*n*** **= 156)**		
**Blood loss total**	94.3 ± 80.1	81.1 ± 67.0	112.2 ± 100.3		94.0 ± 77.8		0.18

The blood supply pattern (single or dual blood supply) or blood vessel diameter was not related to NAC ischemia necrosis grading (Table 4). Combining skin incision type and pattern of the blood vessel, there was no difference in NAC ischemia necrosis risk by either single or double vessel pattern in each type of skin incision (Table 4). Correlations between the pattern or size of the blood vessel with blood loss were also investigated, but there were no correlations found (single versus double, *p* = 0.49) or size of the blood vessel (< 1 mm, = 1 mm, or > 1 mm, *p* = 0.18, Table 4).

A literature review of clinicopathologic risk factors or imaging factors predictive of NAC ischemia necrosis was performed [12, 19, 20, 23, 25–35] and summarized in Table 5, which supported findings derived from the current study.

**Table 5:** Literature review of nipple-areolar complex ischemia necrosis rate and risk factors

Reference	Journal	Publish year	Number	Ischemia(%)	Total necrosis	Risk factor of NAC necrosis
Komorowski et al. [28]	World J Surg	2006	38	5 (13.2%)	3 (7.9%)	Old age (> 45)
Garwood et al. [29]	Ann Surg	2009	64	19 (30%)	N/a	Autologous reconstruction, incision type
			106	14 (13%)	N/a	
Spear et al. [33]	Plast Reconstr Surg	2011	43	N/A	6 (14.0%)	
Algaithy et al. [30]	Eur J Surg Oncol	2012	50	13 (26.0%)	0	Smoking, young age (< 45), incision type, thin areolar flap (< 5 mm)
Carlson et al. [12]	Breast J	2014	71	20 (28.2%)	N/A	Incision site (peri-areolar), operation for cancer (requires additional subareolar excision and frozen section to exclude disease)
Chirappapha et al. [31]	Plast Reconstr Surg Global Open	2014	124	19 (15.3%)	4 (3.5%)	Volume of breast removed
Colwell et al. [32]	Plast Reconstr Surg	2014	482	N/A	21 (4.4%)	Preoperative radiotherapy, implant volume for direct-to-implant, incision type
Bertoni et al. [34]	Ann Surg Oncol	2016	28	N/A	2 (7.1%)	
Bahl et al. [23]	J Am Coll Surg	2016	164	20 (12.2%)	7 (4.27%)	Single blood supply to the breast on MRI
Ahn et al. [11]	Eur J Surg Oncol	2018	220	141 (64.1%)	25 (11.4%)	Age, BMI, existence of ptosis, incision site (peri-areolar), reconstruction
Odom et al. [20]	Plast Reconstr Surg	2018	79	21 (26.5%)	16 (20.2%)	Long operative time, lower whole breast fluorescent intensity, smoking, lower Karnofsky Performance Scale
Daar et al. [25]	Plast Reconstr Surg	2019	4645^a^ (30 studies)	4.62%	2.49%	Incision site (peri-areolar)
Agha et al. [16]	BJS Open	2019	3015^a^ (14 studies)	15.0%	N/a	
Kontos et al. [26]	J Plast Reconstr Aesthet Surg	2020	30	4 (13.3%)	N/A	
Park et al. [35]	Breast	2020	290	45 (15.5%)	25 (8.6%)	Incision site (peri-areolar), decreased tumor-nipple distance, increased breast weight
Webb et al. [19]	Am J Surg	2020	294	105 (35.7%)	0	Increased body mass index
Houvenaeghel et al. [27]	Br J Surg	2021	59	9 (15.0%)	2 (3.0%)	Body weight, body mass index
Lai et al.	Current study	Present	441	Grade 1 29 (6.6%) Grade 2 37 (8.4%)	Grade 3 4 (0.9%)	Age, incision type (peri-areolar), larger breast (specimen > 450 g) MRI: Single or dual blood supply not risk factor for NAC necrosis

^a^Meta-analysis

## Discussion

In the current study, we enrolled 441 NSM procedures with NAC ischemia necrosis grading, clinicopathologic, and MR imaging characteristics to identify risk factors for NAC and/or skin flap necrosis. We found that age, type of skin incision, and larger breast (mastectomy specimen weight) were important risk factors for NAC and/or skin flap necrosis. However, the MRI pattern of blood supply was not a risk factor nor had any correlations with ischemia necrosis of NAC in this study.

Reported studies [12, 25, 30, 35] (Table 5) had shown that the type of skin incisions played important role in the risk of NAC ischemia necrosis. In the current study, the overall NAC ischemia necrosis rate was 9.3%, which included 8.4% partial necrosis (grade2), and 0.9% total necrosis (grade 3, Table 1, Fig. 2) cases. The NAC ischemia necrosis rate was about 6% in the upper outer oblique (radial) incision, 19.6% in the peri-areolar-related incision, and 6.2% in single axillary incision (*p* < 0.01, Table 2). Park et al. [35] had compared three different (inframammary folds (IMF), radial, and peri-areola) incisions of NSM, and the rates of NAC ischemia or necrosis were significantly different. Compared with IMF incisions, the incidence of NAC necrosis in peri-areola incisions is higher. Our findings supported that incision located far away from the areola would decrease the risk of NAC ischemia necrosis [22, 25, 37].

When skin flap or NAC ischemia necrosis was taken as a postoperative event, the single axillary incision (7.4%) was associated with lower NAC or skin flap necrosis than the upper oblique (radial) incision (14.4%) or the per-areolar incision (22.4%, *p* < 0.01, Table 2). As shown in Supplementary File 1, the single axillary incision could prevent disruption of vascular supply to the NAC or skin flap, and the risk of NAC or skin flap ischemia necrosis was expectedly lower (Tables 2 and 3). In the current study, the single axillary incision NSMs were performed with either endoscopic or robotic assistance which were collectively classified as minimal access NSM [36].

Studies have reported that BMI is one of the risk factors (Table 5) for nipple and areola necrosis [38]. BMI is an index of obesity and is highly correlated with breast mastectomy specimen weight. In our previous study [39], we showed that higher BMI women had larger mastectomy specimen weight. In the current study, patients with specimen weights of more than 450 g have a higher (16.9%) incidence of NAC ischemia necrosis than those with less than 180 g specimen weight (4.3%, odds ratio = 4.6, *p* = 0.03, Table 3). Some studies suggested that the increase in BMI will increase the operation time of NSM, thereby increasing the possibility of nipple necrosis [19, 40, 41]. In the current study, *BMI* ≥ 27 was associated with a trend of increased NAC or skin flap necrosis rate (19.3%) compared with patients whose BMI was < 27 (11.2%, *p* = 0.08). Some studies also reported that the surgeon’s experience will affect the incidence of NAC necrosis, and surgical delay [34, 42], in which the sub-nipple tissue and skin flap were divided with delayed NSM 2 weeks later would also decrease the risk of NAC ischemia necrosis.

Breast MRI had been one of the important imaging evaluation tools for preoperative breast cancer patients [39, 43–45]. Blood supply of the NAC could be predictive of NAC necrosis after surgery [23]. In a previously reported study [23], patients with MRI features of a single blood vessel pattern had a higher risk of NAC necrosis than a double blood vessel. However, in the current study, we did not observe a difference in NAC ischemia necrosis rate between patients with single or double vessel supply as determined by preoperative breast MRI (Table 4).

However, the authors found that blood vessel diameter was related to ischemia necrosis of NAC. The NAC ischemia necrosis rate was highest in the MRI vessel diameter of 1 mm (17.8%), followed by 9.9% in the vessel > 1 mm diameter and lowest (1.8%) in the vessel < 1 mm (Table 4). We further analyzed the impact of single or dual blood vessel supply to different types of skin incisions and found no difference in blood vessel pattern to the rate of NAC necrosis in either type of skin flap incisions (Table 4). Whether preoperative breast MRI blood vessel pattern could be informative or predictive for NAC ischemia necrosis [23] remained unclear due to rare and inconsistent results.

Our current study is limited by its retrospective nature and the small number of NSM procedures analyzed which could lead to bias in outcomes interpretation. The skills and experience of surgeons could also affect the risk of NAC necrosis [11]; in the current study, most of the NSM procedures were performed by the principal investigator (HWL), which could exclude surgeon-related bias. Braun et al. [46] reported that after NSM, breast reconstruction methods also affect the necrosis rate of NAC; however, in the current study, we did not find breast reconstruction as a risk factor. Despite these limitations, our studies enrolled 441 NSM procedures with detailed clinicopathologic factors and validated postoperative skin flap or NAC survival status to evaluate risk factors for NAC or skin flap necrosis. We also have 270 patients with preoperative breast MRI to validate the implication of MRI vessel pattern on NAC ischemia necrosis post-NSM. Therefore, the results and information derived from the current study are valuable.

## Conclusion

This retrospective study examined both clinical and imaging risk factors for NAC necrosis. Our current study ascertained that certain risk factors, like the type of skin flap (peri-areolar) incisions, age, larger breast (mastectomy specimen weight > 450 g), played an important role in ischemic necrosis of NAC or skin flap in patients post NSM. Avoiding peri-areolar incision and appropriate patient selection such as younger age, *BMI* < 27, and the vessel < 1 mm on preoperative breast MRI may greatly decrease the risk of NAC necrosis. We did not find the pattern of blood vessels (single versus double) around the NAC to be related to ischemia necrosis of the NAC. Larger retrospective or future prospective studies are needed to validate this hypothesis.

## Supplementary Information

**Additional file 1: Supplementary File 1.** Illustration of types of skin incision used in the current study. (a) upper outer incision (radial incision). (b) peri-areolar incision (with or without axillary incision). (c) single axillary incision. (d) infra-mammary +axillary incisions.

## Data Availability

All the data used in the current study could be available after the permission of principle investigator (Hung-Wen Lai) by request.
