# Supplementary figures and images for: Nipple-areolar complex (NAC) or skin flap ischemia necrosis post nipple-sparing mastectomy (NSM)—analysis of clinicopathologic factors and breast magnetic resonance imaging (MRI) features
Source: World J Surg Oncol. 2023 Jan 25;21:23. doi: 10.1186/s12957-023-02898-x (PMC9875411; doi:10.1186/s12957-023-02898-x)

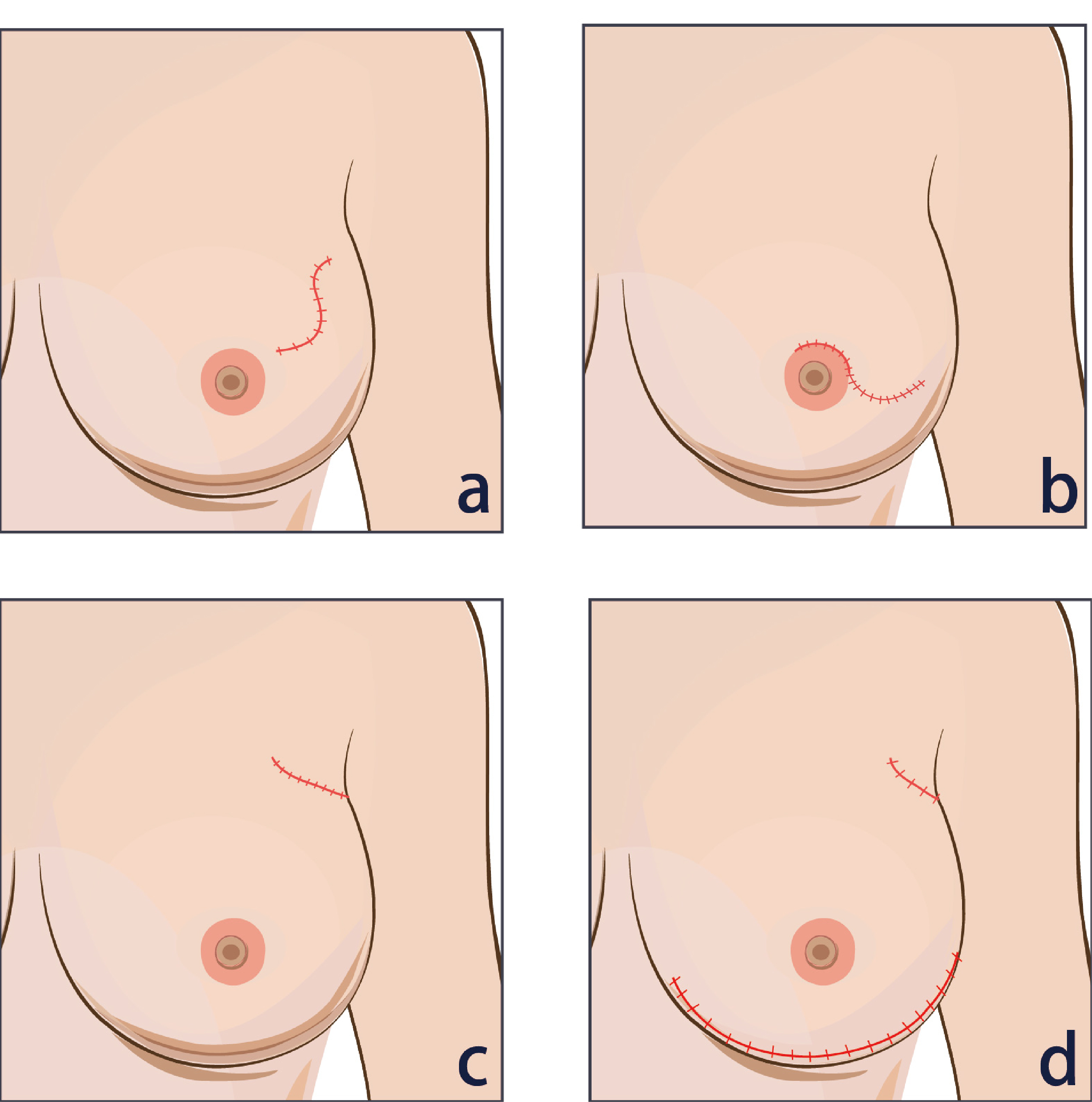

Supplement: Supplementary file 1 — Additional file 1: Supplementary File 1. Illustration of types of skin incision used in the current study. (a) upper outer incision (radial incision). (b) peri-areolar incision (with or without axillary incision). (c) single axillary incision. (d) infra-mammary +axillary incisions. [file 12957_2023_2898_MOESM1_ESM.jpg]
